# Urchin-like magnetic microspheres for cancer therapy through synergistic effect of mechanical force, photothermal and photodynamic effects

**DOI:** 10.1186/s12951-022-01411-y

**Published:** 2022-05-12

**Authors:** Kai Wu, Ali Mohsin, Waqas Qamar Zaman, Zefei Zhang, Wenyan Guan, Maoquan Chu, Yingping Zhuang, Meijin Guo

**Affiliations:** 1 State Key Laboratory of Bioreactor Engineering, East China University of Science and Technology, 130 Meilong Road, P.O. Box 329#, Shanghai, 200237 People’s Republic of China; 2 Biomedical Multidisciplinary Innovation Research Institute and Research Center for Translational Medicine at Shanghai East Hospital, School of Life Sciences and Technology, Tongji University, Shanghai, 200092 People’s Republic of China; 3 Institute of Environmental Sciences and Engineering, School of Civil and Environmental Engineering, National University of Sciences and Technology (NUST), Sector H-12, Islamabad, 44000 Pakistan; 4 Materials and Biomaterials Science and Engineering, University of California, Merced, CA 95343 USA

**Keywords:** Urchin-like hollow magnetic microspheres, Laryngocarcinoma therapy, Magneto-mechanic force, Photothermal effect, Photodynamic effect

## Abstract

**Background:**

Magnetic materials mediated by mechanical forces to combat cancer cells are currently attracting attention. Firstly, the magnetic force penetrates deeper into tissues than the NIR laser alone to destroy tumours. Secondly, the synergistic effect of nano-magnetic-material characteristics results in a viable option for the targeted killing of cancer cells. Therefore, mechanical force (MF) produced by magnetic nanomaterials under low frequency dynamic magnetic field combined with laser technology is the most effective, safe and efficient tool for killing cancer cells and tumour growth.

**Results:**

In this study, we synthesized novel urchin-like hollow magnetic microspheres (UHMMs) composed of superparamagnetic Fe_3_O_4_. We demonstrated the excellent performance of UHMMs for killing laryngocarcinoma cancer cells through mechanical force and photothermal effects under a vibrating magnetic field and near-infrared laser, respectively. The killing efficiency was further improved after loading the synthesised UHMMs with Chlorin e6 relative to unloaded UHMMs. Additionally, in animal experiments, laryngocarcinoma solid tumour growth was effectively inhibited by UHMMs@Ce6 through magneto-mechanic force, photothermal and photodynamic therapy.

**Conclusions:**

The biocompatibility and high efficiency of multimodal integrated therapy with the UHMMs prepared in this work provide new insights for developing novel nano therapy and drug loading platforms for tumour treatment. In vivo experiments further demonstrated that UHMMs/Ce6 are excellent tools for strongly inhibiting tumour growth through the above-mentioned characteristic effects.

**Graphical Abstract:**

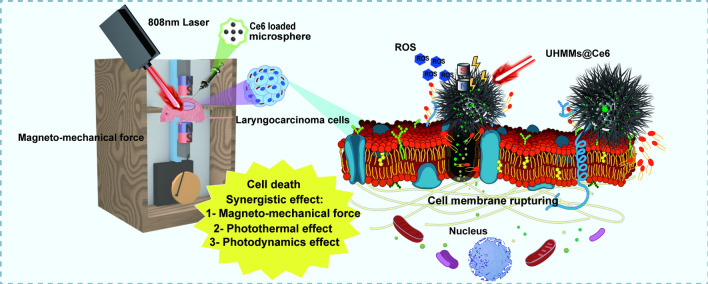

**Supplementary Information:**

The online version contains supplementary material available at 10.1186/s12951-022-01411-y.

## Background

Cancer is a fatal disease in China and globally, as evidenced by a growing trend in morbidity and mortality year after year. Laryngocarcinoma is considered a malignant and lethal type of tumour [1]. Nowadays, surgical treatment has achieved good results in the resection of early laryngocarcinoma cancer over the years [2]. However, the large-scale resection has also caused irreversible damage to the human body [3] and is no longer applicable to advanced laryngocarcinoma cancer [4]. Additionally, other conventional cancer treatments such as radiation therapy and chemotherapy, owing to inadequate selectivity, toxicity to surrounding healthy cells and drug resistance in cancers, can lead to more complications and adverse reactions to the human system [5–7]. Due to the limitations and insecurities of present tumour treatment methods, exploring safe and effective therapy is highly desired.

Recently, magnetic iron oxide nanoparticles (IONPs) with unique sizes and physical properties have been widely applied in biomedical engineering because of their excellent biocompatibility [8–11] with bioimaging [12, 13], diagnostics [14], therapeutics [15–21] and as drug carriers [22, 23]. These magnetic IONPs have already been approved by the United States Food and Drug Administration for clinical applications [24]. Because magnetic IONPs with different morphology and nano-size could be easily prepared [25], more features have been explored in biomedical applications in recent years, like Fenton reaction and hyperthermia under magnetic field or near-infrared (NIR) laser. Although some remarkable results had been achieved in cancer treatment when using Fenton reactions to generate hydroxyl radicals (OH^•^) in the tumour microenvironment (TME) [26–28], the effects and applications were limited by the insufficient acidity of TME (the optimum pH for Fenton reactions is between 2.0 and 4.5 [29], but the pH of TME is approximately 6.5 [30, 31]).

Hyperthermia with magnetic IONPs is an alternative and promising approach for cancer treatment. Cancer cells are more sensitive to the temperature ranging from 40 to 48 °C than the normal cells [32], inferring that cancer cells at tumour site can be heat-killed using magnetic hyperthermia therapy (MHT) [16] or photothermal therapies (PTT) [15, 33] generated by magnetic IONPs. Compared to large-scale resection of surgery and lacking-selectivity of chemotherapy [4–7], both MHT & PTT show more accurate and better effects at the local tumour site, causing less damage to the human body. However, both therapies have their drawbacks. Previously, several reports have shown that the penetration depth of near-infrared laser was low when using different bio-samples with NIR illumination. It was about 10 mm penetration depth with porcine muscle tissues when using 808 nm laser [34–37], indicating that the near-infrared laser employed in photothermal therapy has great constraints for irradiating magnetic IONPs in deep depth tissues [38]. Compared with PTT, deep penetration problems can be addressed effectively with MHT, but the heating yield per nanoparticle amount of MHT is much inferior to PTT [38–40]. Additionally, MHT requires high frequencies alternating magnetic fields ranging from kilohertz to megahertz [41–44] and appropriate amplitude [38], which is costly and inconvenient.

Due to the good magnetic response-ability of IONPs in a magnetic field, the magneto-mechanical force produced by magnetic IONPs under low frequency dynamic or alternating magnetic fields has currently attracted much attention of researchers. It provides a new strategy for the remote, non-invasive and precise treatment of cancers. Firstly, a low frequency dynamic or alternating magnetic field is very easy to build and has little effect on the human body. Secondly, magnetic IONPs could display movement, oscillate or rotate behaviour under low-frequency vibrating magnetic fields and generate mechanical force to stimulate cancer cells to induce cell apoptosis or death [44–47]. Lastly, compared with NIR, the magnetic field could penetrate deeper tissues and affect magnetic particles' behaviour inside tissues, stimulating deeper into tumour and destroying it. The manipulation of tumour apoptosis induced by mechanical force comprises three pathways [48]: (1) activating ion channels to manipulate intracellular ion flux [49], (2) acting directly on death receptors to initiate apoptosis [50, 51], and (3) entering cells and destroying the lysosome or mitochondrial membranes [47, 52, 53]. Kim et al. [54] synthesised magnetic nano-disks that are modifiable with special antibodies and move or rotate under an alternating magnetic field to achieve in vitro cancer-cell destruction of approximately 90%. The vibrating magnetic field used in this magneto-mechanical actuation technique is usually only a few tens of Oersteds with a frequency of less than 100 Hz [44] and the mechanical force for manipulating tumour evolution only requires a pico-Newton (*pN*) level [55, 56], which has many advantages including safety, less cost and easy handling.

Although there are many advantages of magneto-mechanical force; however, as a mono-modality treatment, the cancer cells' killing effect is not sufficient. At the same time, it is a good strategy to kill cancer cells by the combinatorial effect of different treatments as presented in many works currently [28, 57–60]. Therefore, combining the deep penetration of magneto-mechanic force and the accuracy of photothermal effect from magnetic IONPs is worth exploring in cancer treatment.

Magnetic IONPs with a regular shape, like spheres [18, 61–65], are commonly used in cancer treatment. However, herein we use a template-assisted hydrothermal route with a calcination method to prepare structurally versatile, novel urchin-like hollow magnetic microspheres (UHMMs) of Fe_3_O_4_. The diameter of the UHMMs is approximately 800 nm, and the average length of the Fe_3_O_4_ rods is approximately 400–600 nm. Compared to regular magnetic IONPs, the Fe_3_O_4_ nanorods from UHMMs in our work can increase the stimulation of mechanical force to cells [66, 67] and improve the photothermal conversion ability under near-infrared light because of surface plasmon resonance [68, 69]. The synthesised nanoparticles exhibited good magnetism, with a saturation magnetisation of 62.70 emug^−1^, and calculated photothermal conversion efficiency of 16.50%. Simultaneously, to improve the cancer cells killing effect, the hollow structure of UHMMs was used to load a photosensitiser (Chlorin e6, Ce6) that could generate reactive oxygen species (ROS) to induce cancer cells apoptosis at the NIR region [70, 71]. The Ce6 loading rate of UHMMs could reach to 11.51% (w/w). Upon loading with Ce6, the prepared UHMMs generate mechanical force even when applying a low-frequency vibrating magnetic field (VMF) and trigger photothermal and photodynamic effects with an 808 nm laser. Laryngocarcinoma cancer cells were effectively killed in vitro, and solid tumour growth was effectively inhibited in vivo. The safety and high efficiency of multimodal integrated therapy with UHMMs prepared in this work provide new insights for developing novel nano therapy and drug loading platforms for tumour treatment.

## Results

### Preparation and characterisation of UHMMs

**Scheme 1:**
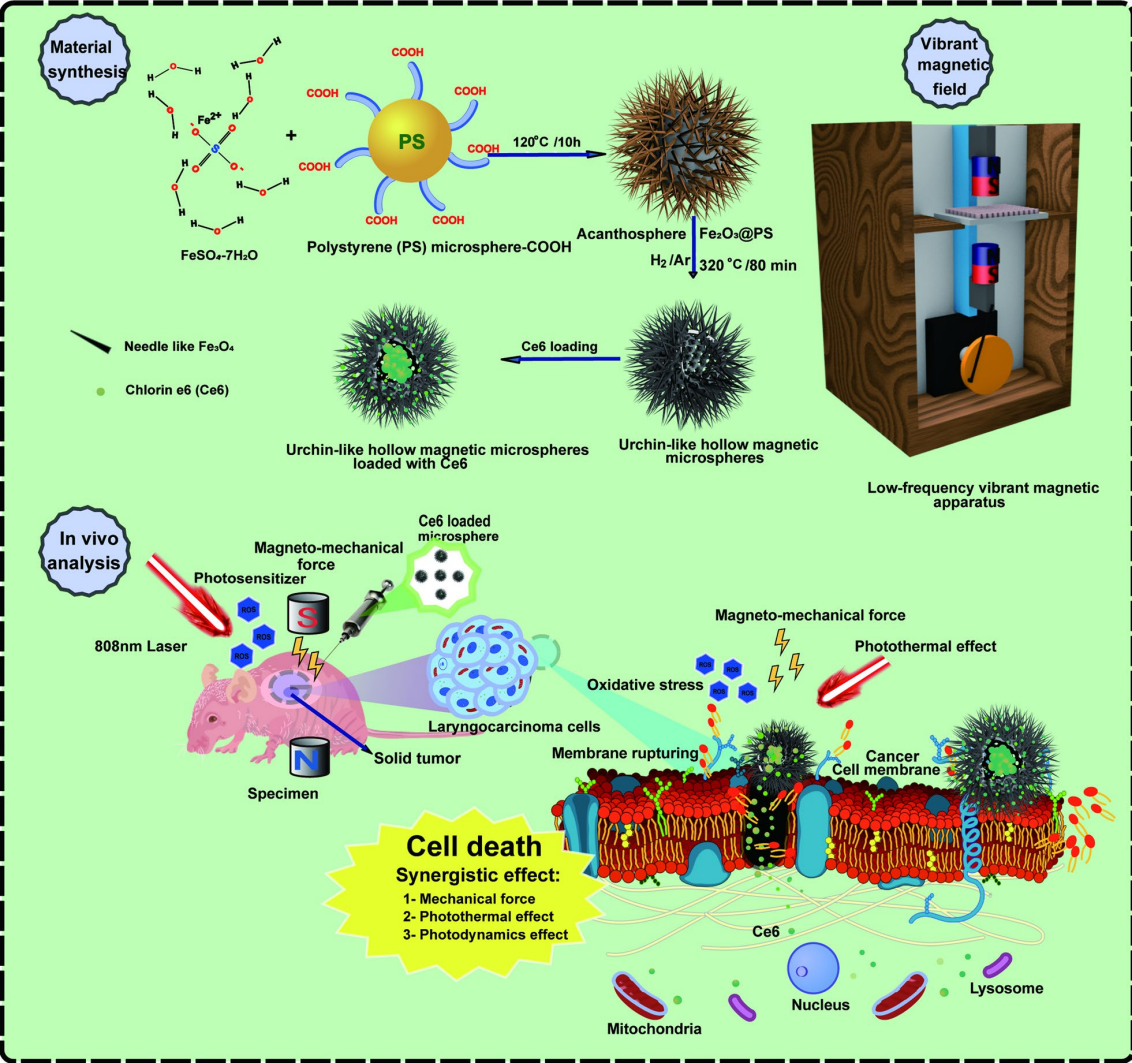
Schematic illustration of the preparation of urchin-like hollow magnetic microspheres (UHMMs) and cancer killing mechanisms

UHMMs were fabricated through a two-step process, in which carbonylated polystyrene (PS) microspheres and FeSO_4_7H_2_O were used to synthesise the UHMM precursor using the hydrothermal method. The UHMMs precursor has a core–shell nanostructure that comprises Fe_2_O_3_ nanorods as the shell and PS as the core. The PS microspheres served as templates and were removed when Fe_2_O_3_@PS was transferred to Fe_3_O_4_ during heat treatment in a tube furnace under an Ar/H_2_ (5%) atmosphere. Scheme 1 shows a schematic illustration of the UHMMs synthesis.

**Fig. 1:**
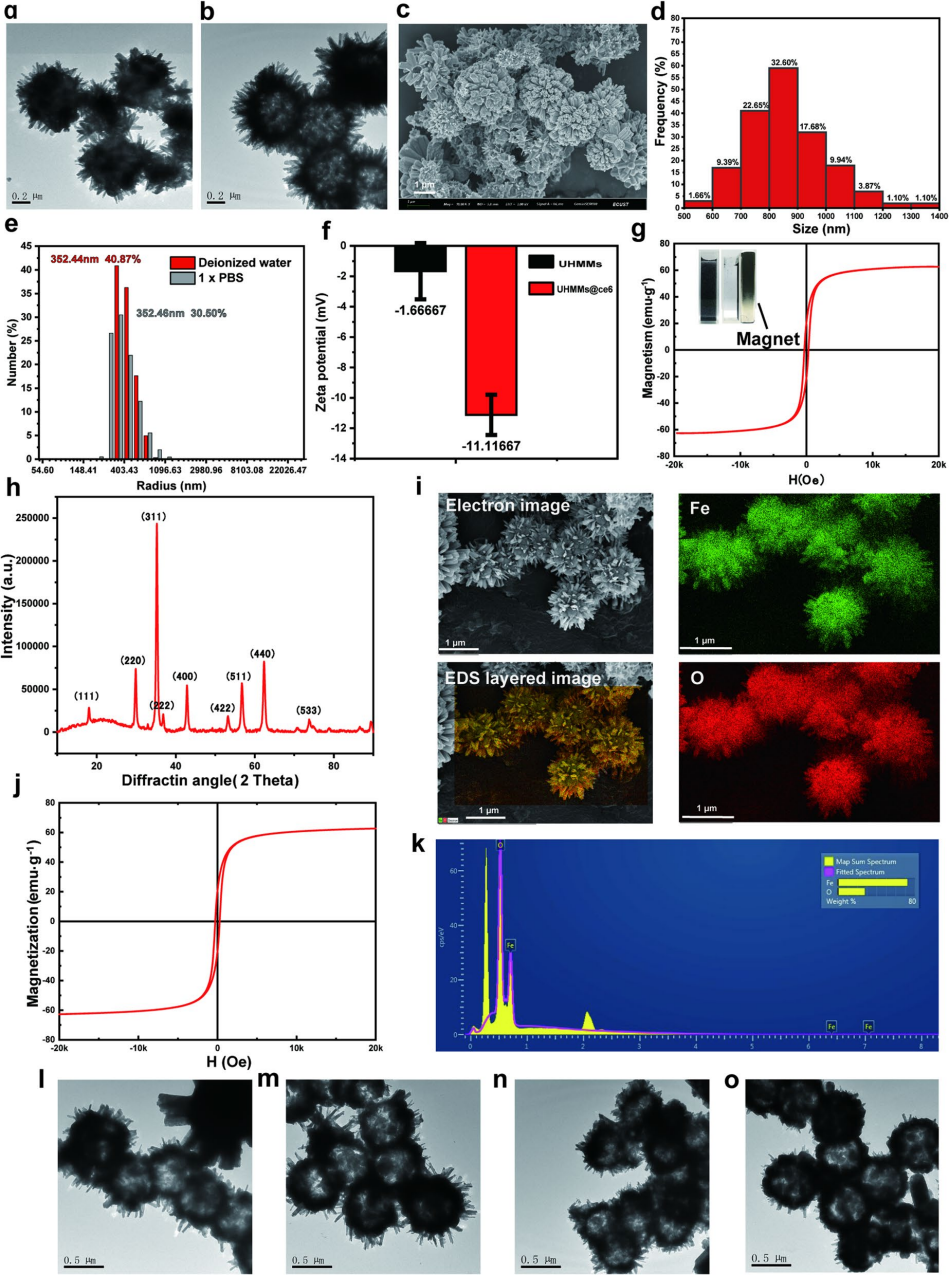
UHMMs characterisation. **a** image of Fe_2_O_3_@PS microspheres (TEM). **b**, **c** UHMMs images (TEM, SEM). **d**, **e** UHMMs particle size distribution with TEM and DLS measurement. **f** zeta potential of the UHMMs and UHMMs@Ce6. **g** UHMMs hysteresis loops with SQUID-VSM. **h** UHMMs pattern with XRD. **i**, **k** images of EDS and element mapping. **j** UHMMs magnetism stability after 3 months. **l**, **m** UHMMs structure stability after 1 d and 30 d of storage 3 months. **n**, **o** UHMMs structure stability after treated by a low frequency vibrating magnetic field for 2 and 10 h

Figure 1a shows the morphology of the precursor-Fe_2_O_3_@PS microspheres under a transmission electron microscope (TEM). The TEM images revealed that the precursor possessed a core–shell structure; Fe_2_O_3_ nanorods were observed on the surface of carboxy PS due to the chelation reaction between Fe^2+^ and -COOH. The diameter of the PS core was 500 nm, and the length of the Fe_2_O_3_ nanorods was approximately 400 nm. Additionally, the temperature or heating time during the hydrothermal reaction manipulated the density and length of the Fe_2_O_3_ nanorods through material concentration (Additional file 1: Fig. S1a–i). After hyperthermia and reduction, the PS template was removed to obtain ~ 800 nm UHMMs (Fig. 1b, c); UHMMs have a uniform morphology with a hollow urchin-like structure. One hundred eighty-three particles from the TEM images were taken for the calculation. Figure 1d shows the distribution of the UHMMs particle size; most of the particles (83.98%) were smaller than 1000 nm, and 32.6% particles measured 800–900 nm, which corresponded with the result of DLS measurement in different solutions (Fig. 1e, Table 1 and Additional file 1: Fig. S4a, b).

**Table 1:** UHMMs particle size distribution with DLS measurement in deionized water and 1 × PBS (concentration: 0.1 mg/mL)

Solution	Peak	Diameter (nm)	Diffusion Coefficient (cm^2^/s)	Pd (%)	Pd (nm)	Pd Index	Intensity (%)	Mass (%)	Number (%)
Deionized water	Peak 1	887.8	5.40×10^–9^	22.9	101.61	0.05	69	69.1	99.9
	Peak 2	16,702.4	2.90×10^–10^	56.8	4746	0.32	31	30.9	0.1
1 × PBS	Peak 1	829.1	5.80×10^–9^	36.1	149.69	0.13	53.3	53.4	99.8
	Peak 2	14,652.3	3.30×10^–10^	66.3	4860.71	0.44	46.7	46.6	0.2

The UHMMs structure corresponds to the set temperature and heating time. For instance, the hollow structure was disrupted by increasing the temperature above 400 °C or by overtime heating (Additional file 1: Fig. S2e and l). However, acanth sphere clusters formed at temperatures below 290 °C or elapsed times of less than 1 h due to PS melting (because the PS was not eradicated) (Additional file 1: Fig. S2b and i). Therefore, optimal annealing conditions to prepare UHMMs with good morphology and structure were 320 °C and 80 min in a tube furnace (Fig. 1c).

X-ray diffraction (XRD) patterns of the UHMMs show that the diffraction peaks were assigned to the (111), (220), (311), (222), (400), (422), (511), (440), and (533) planes of the Fe_3_O_4_ criterion card (JCPDS19-0629) (Fig. 1h). Therefore, UHMMs are highly pure Fe_3_O_4_ with a spinel structure and possess good superparamagnetic properties with a magnetisation saturation rate of 62.72 emu·g ^−1^ (Fig. 1g). The O and Fe peaks are present in the energy dispersive spectroscopy (EDS) spectrum (Fig. 1i and k), confirming that UHMMs contain only two elements. The atomic proportions of Fe and O are 72.18% and 27.83%, respectively, (Fig. 1k and Table 2) confirming the XRD analysis results. The measured zeta potential of the UHMMs was − 1.67 mV; the UHMMs@Ce6 zeta potential of − 11.12 mV is attributed to the Ce6 from the abundant carboxyl groups (Fig. 1f).

**Table 2:** The atomic proportions of Fe and O in UHMMs

Element	Line type	Wt (%)	Atomic (%)
O	K series	27.82	57.37
Fe	L series	72.18	42.63
Total		100.00	100.00

To confirm the good structural stability and magnetism of the UHMMs, we maintained the UHMMs solution (0.5 mg/mL in deionised water) for 1 d, 30 d, and 3 months at room temperature (25 °C). The solution was also placed in a low-frequency vibrating magnetic field for 2 h and 10 h. The UHMMs maintained their original structure after 1 d and 30 d of storage (Fig. 1l, m); additionally, the magnetisation saturation was maintained at 62.70 emu g^−1^ (Fig. 1j) after 3 months. Therefore, the synthesised UHMMs possessed good structural and magnetic stability. The UHMMs later treated by a low-frequency vibrating magnetic field for 2 h and 10 h showed similar structural and magnetic results; thus, low-frequency vibrating magnetic fields cannot destroy the UHMMs structure (Fig. 1n, o).

The above-presented results indicate that the UHMMs synthesised in this study have ideal and steady urchin-like structures and magnetisms and can be potentially applied to cancer cell destruction with mechanical force induced by a low-frequency vibrating magnetic field.

**Fig. 2:**
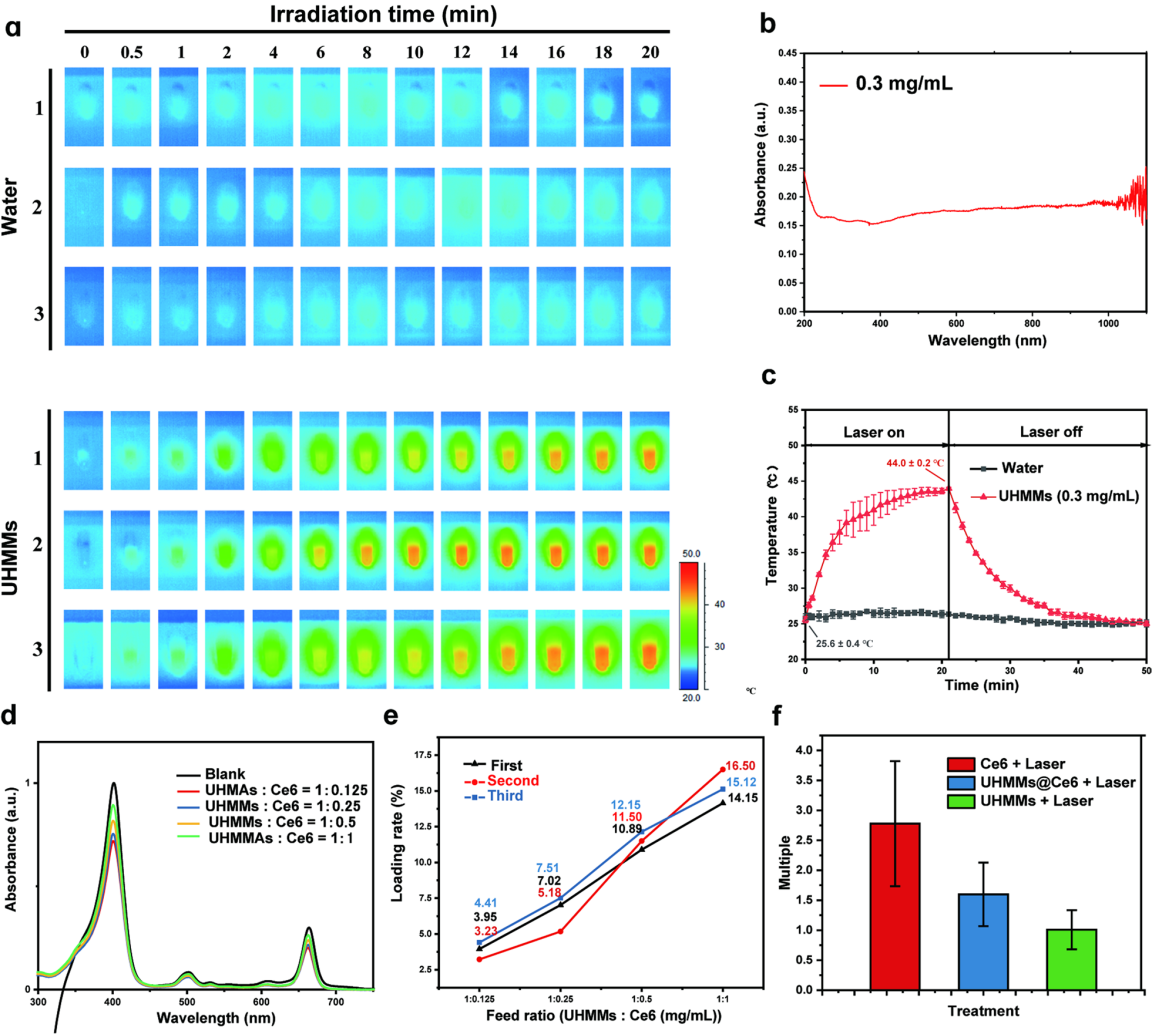
**a** Infrared thermal images of water and UHMMs dispersion under an 808 nm laser. **b** UHMMs absorption spectrum. **c** mean UHMMs dispersion temperature profile from the infrared thermal images in Fig. 3a under the 808 nm NIR laser. **d**, **e** scheme of Ce6 loaded to UHMMs. **f** ROS level of UHMMs@Ce6 under the 808 nm laser

The photothermal conversion efficiency is closely related to the absorption coefficient at a specific wavelength. Therefore, Fig. 2b shows the absorption spectrum of 0.3-mg/mL UHMMs aqueous dispersions in the ultraviolet, visible, and near-infrared regions. The absorption coefficient (*E*) of the UHMMs aqueous dispersions (Fig. 2b) is 6.12 at 808 nm and calculated using Eq. (1):

1 $${\varvec{E}}=\frac{{\varvec{A}}{\varvec{\lambda}}}{{\varvec{c}}\cdot {\varvec{l}}}$$

where *E* is the absorption coefficient, *Aλ* is the absorption value of the UHMMs aqueous dispersions at 808 nm, *c* is the concentration of the UHMMs aqueous dispersions, and *l* is the thickness of the cuvette.

Because UHMMs are structured on Fe_3_O_4_ nanorods with good absorption at 808 nm, we used an 808 nm laser (0.38 W, 0.35 cm^2^) to irradiate the UHMMs aqueous dispersions (0.3 mg/mL) for 20 min and used an infrared thermal imager to collect the temperature data and images (Fig. 2a and c). After the initial 7 min of irradiation, the temperature of the UHMMs aqueous dispersions increased significantly from 25.6 °C ± 0.4 °C to 39.6 °C ± 2.4 °C. Subsequently, the temperature profile plateaued after 10 min and reached 44.0 °C ± 0.2 °C at 20 min. We obtained the photothermal conversion efficiency of UHMMs with the temperature-drop curve after removal of the 808 nm laser using Eq. (2):

2 $${\varvec{\eta}}=\frac{{\varvec{h}}{\varvec{A}}\left({{\varvec{T}}}_{{\varvec{m}}{\varvec{a}}{\varvec{x}}}-{{\varvec{T}}}_{{\varvec{s}}{\varvec{u}}{\varvec{r}}{\varvec{r}}}\right)-{{\varvec{Q}}}_{{\varvec{i}}{\varvec{n}},{\varvec{s}}{\varvec{u}}{\varvec{r}}{\varvec{r}}}}{{\varvec{I}}(1-{10}^{-{\varvec{A}}{\varvec{\lambda}}})}$$

where *η* is the conversion efficiency calculated using the temperature-drop curve, and *hA* is the coefficient of the photothermal conversion efficiency. (*T*_*max*_* − T*_*surr*_) was 18.94 °C (Fig. 2c); *Q*_*in,surr*_ = *C*_*p*_*m*_*water*_ (*T*_*max*_* − T*_*surr*_); *I* is the power density of the 808 nm laser, and Aλ is the absorption value of UHMMs aqueous dispersions (0.3 mg/mL) at 808 nm.

Therefore, *η* was calculated as 16.50%. The control group, which used deionised water to increase the temperature from 25.8 ℃ ± 0.6 under the same conditions (Fig. 2a and c), simultaneously indicated the safety of the power of the 808 nm laser applied in our work. Furthermore, an 808 nm laser was used to re-irradiate the UHMMs solution after cooling to room temperature for three cycles; the UHMMs retained excellent photothermal efficiency from an initial ~ 25.0 °C to ~ 44 °C. Thus, the UHMMs have good photothermal conversion efficiency and stability for application in tumour photothermal therapy (PTT).

To further enhance the killing effect of UHMMs in tumour therapies, Chlorin e6 (Ce6), a photosensitiser that kills cancer cells by generating cytotoxic reactive oxygen species (ROS) under light activation was selected. A little amount of Ce6 was loaded into the UHMMs using the sedimentation method at a concentration of 1 mg/mL in ethanol solution. Different feed ratios between the UHMMs and Ce6 were selected for loading analysis (Fig. 2d). After analysis using an ultraviolet spectrophotometer and calculation from Eq. (3), the average loading rate was found to be approximately 11.51% (w/w), with a feed ratio of 1:0.5 (UHMMs: Ce6 (Fig. 2e).

3 $$\frac{{{\varvec{A}}{\varvec{\lambda}}}_{{\varvec{b}}}-{\varvec{A}}{{\varvec{\lambda}}}_{{\varvec{l}}}}{{\varvec{A}}{{\varvec{\lambda}}}_{{\varvec{b}}}}\times \frac{{{\varvec{c}}}_{{\varvec{C}}{\varvec{e}}6}}{{{\varvec{c}}}_{{\varvec{U}}{\varvec{H}}{\varvec{M}}{\varvec{M}}{\varvec{s}}}}\times 100\boldsymbol{\%}$$

where *Aλb* is the optical density of the Ce6 blank at 400 nm; *Aλl* is the optical density of Ce6 in the supernatant after the loading steps; and *C*_*Ce6*_ and *C*_*UHMMs*_ are the concentrations of Ce6 and UHMMs in the sedimentation system, respectively.

To verify the successful Ce6 drug loading on UHMMs, we employed reactive oxygen fluorescent probe (DCFH-DA) to detect the ROS level of the UHMMs@Ce6, UHMMs, and Ce6 in cells (maintaining the concentration of UHMMs@Ce6, UHMMs at 0.75 mg/mL, and Ce6 at 86.25 μg/mL under light exposure for 10 min). Figure 2f shows the difference in reactive oxygen among the Ce6, UHMMs@Ce6, and UHMMs after exposure to the 808 nm laser. As expected, the amount of ROS in UHMMs@Ce6 was 1.60 ± 0.53 times higher than that in the empty UHMMs but less than that in the Ce6 group due to the cancer cells killed by photothermal irradiation with the 808 nm NIR laser. Thus, UHMMs load Ce6 and produce a weak ROS under an 808 nm NIR laser for application in PDT tumour therapy.

### In vitro toxicity of UHMMs and UHMMs@Ce6

**Fig. 3:**
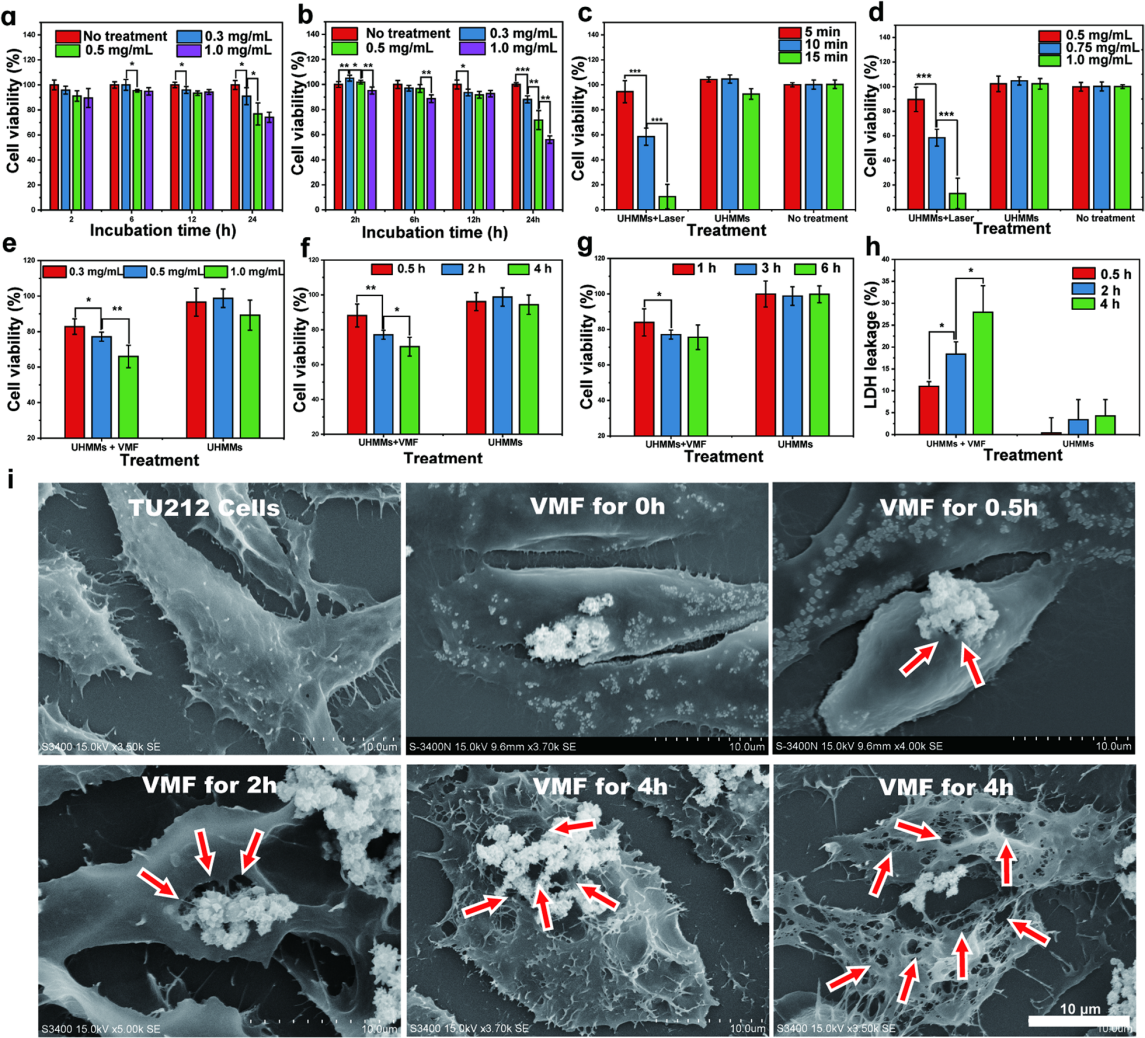
**a** In vitro toxicity of UHMMs. **b** In vitro toxicity of UHMMs@Ce6. **c** Cancer cell viability after in vitro treatment with an 808 nm laser and different irradiation times. **d** Cancer cell viability after in vitro treatment with an 808 nm laser with different UHMMs doses. **e** Cancer cell viability after in vitro treatment with VMF and different UHMMs doses. **f** Cancer cell viability after in vitro treatment with VMF and different vibration time. **g** Cancer cell viability after in vitro treatment with VMF with different incubation times. **h** LDH leakage after VMF treatment and different times. **i** SEM images: morphology of TU212 cells with UHMMs after VMF treatment for 0, 0.5, 2, and 4 h

Estimating the toxicity of UHMMs before performing subsequent experiments in vitro is imperative to guarantee safety. Firstly, laryngocarcinoma cancer cells (TU212) were incubated with RPMI1640 culture solution containing UHMMs/UHMMs@Ce6 with low to high concentrations (0.3, 0.5, and 1.0 mg/mL, PBS-dispersed) for 2, 6, 12, and 24 h. Second, we evaluated cell viability through a Cell Titer-Glo® assay, which detects the number of viable cells by quantitative determination of ATP with luminescence. Figure 3a, b show the toxicities of UHMMs and UHMMs@Ce6, respectively. At low concentration (0.3 mg/mL), TU212 cells maintained good viability for 2–24 h; 91.01% ± 6.86 and 88.08% ± 2.80 of cancer cells survived UHMMs and UHMMs@Ce6 incubation for 24 h, respectively.

However, for an increased PBS-dispersed UHMMs/UHMMs@Ce6 concentration of 0.5 mg/mL and prolonged incubation time of 24 h, the viability of the cells significantly decreased compared to the untreated group; 76.84% ± 8.77 and 71.57% ± 7.55 of cancer cells survived in the UHMMs and UHMMs@Ce6, groups, respectively. The cancer cell viability in the control group (no materials) was ~ 100% after 24 h of incubation.

The cancer cell viability decreased remarkably at UHMMs/UHMMs@Ce6 concentrations of 1 mg/mL; 74.16% ± 4.10 (UHMMs) and 55.99% ± 2.94 (UHMMs@Ce6) of cancer cells survived after 24 h of incubation. These results indicate that the toxicity of UHMMs@Ce6 was greater than that of UHMMs at the same concentration for a longer incubation time.

In addition, the L929 cell line, one of the important constituent cells of the dermis, was also tested in toxicity experiments and the obtained results were same as expected. The cells kept a good viability after co-cultured with low concentration of UHMMs and UHMMs@Ce6 dispersion in 24 h. Similarly, a high concentration of UHMMs and UHMMs@Ce6 dispersion would not be friendly to cells with long time co-culturing. Additionally, compared with UHMMs, the results showed greater toxicity with UHMMs@Ce6 (Additional file 1: Fig. S3a, b).

Thus, UHMMs/UHMMs@Ce6 possess weak toxicity to cancer cells at lower concentrations during a short incubation time (< 12 h) but show non-negligible toxicity at higher concentrations and longer incubation times, especially for UHMMs@Ce6. Part of the toxicity is attributed to the Fenton reaction of Fe_3_O_4_, which releases Fe^2+^ and Fe^3+^ into the cell culture medium. Ce6 also showed little phototoxicity to cells during incubation.

### Cancer cell killing efficiency of UHMMs mediated by single mode therapy

UHMMs possess good photothermal conversion efficiency. Therefore, to evaluate their photothermal damage effect, TU212 cells were incubated with UHMMs-RPMI1640 culture medium solution and irradiated with an 808 nm laser (1.08 W/ cm^2^). The viability of the TU212 cells treated by "UHMMs + laser" was associated with the irradiation time and the concentration of PBS-dispersed UHMMs. Firstly, the concentration of PBS-dispersed UHMMs was maintained at 0.75 mg/mL. Figure 3c shows the viability of TU212 cells after treatment with the 808 nm laser for 5, 10, and 15 min; 10.44% ± 9.80 cells survived after 15 min of irradiation, and the cell viabilities of the remaining two groups was 94.49% ± 8.79 (5 min) and 58.49% ± 6.85 (10 min). Secondly, keeping the irradiation time of 808 nm laser for 10 min, 89.61% ± 9.93, 58.49% ± 6.85, and 13.12% ± 12.42 of cancer cells retained activity at 0.5, 0.75, and 1.0 mg/mL UHMMs dispersions, respectively (Fig. 3d).

These results indicate the concentration dependency of UHMMs when used for photothermal effects to damage cancer cells. The group of "RPMI1640 + laser" and "UHMMs (no laser)" had no apparent adverse effect on cell viability, as both showed high and regular cell activity during the experiments.

Hoechst/Propidium Iodide (PI) fluorescence double staining qualitative detection also indicates the damage to cancer cells corresponding to the UHMMs concentration of the irradiation time (Additional file 1: Fig. S6 and S7). The dead cells are shown as red because the cell membrane lost biological functions and PI stained cell nucleus; contrarily, the survived cells are shown as blue. Thus, the dead cell (red colour) count was increased with the increased UHMMs concentration or irradiation time. These results confirm that UHMMs have good photothermal efficiency for killing cancer cells using 808 nm laser and promising tumour photothermal therapy applications.

A simple device (Additional file 1: Fig. S5) with two permanent magnets was developed to generate a low-frequency vibration, which is a magnetic field that can produce a sine vibration. The details of the working principle of the low-frequency vibration magnetic field generator can be found in Experimental Section and work reported in previous study [44]. Herein, we designed three groups to explore the cancer cell-killing effects of UHMMs in VMF with different incubation times, vibration times, and dispersion concentrations. TU212 cells were incubated with different concentrations of UHMMs (0.3, 0.5, and 1.0 mg/mL, RPMI1640 medium) for 1, 3, and 6 h and exposed to VMF for 0.5, 2, and 4 h. As expected, the cancer cell killing efficiency of UHMMs with VMF was significantly greater than that of the untreated and control groups. Figure 3e demonstrates the cancer cell killing efficiency when co-cultured with UHMMs in VMF in different situations. Remarkably, the killing efficiency was correlated with the UHMMs concentration or the vibration time; 17.18% ± 4.37%, 22.89% ± 2.55%, and 34.00% ± 6.32% of cancer cells were killed when the UHMMs concentration increased from 0.3–1.0 mg/mL during 2 h of vibration. Further, 11.84% ± 6.57%, 22.00% ± 2.55%, and 29.00% ± 5.53% of cancer cells were killed (efficiency was positively correlated with treatment time) for vibration time increased from 0.5–4 h using 0.5 mg/mL UHMMs (Fig. 3f).

Figure 3g shows viable cell activities of 84.00% ± 7.63%, 77.11% ± 2.55%, and 75.60% ± 6.90% when incubated with UHMMs for 1, 3, and 6 h, respectively when the concentration of UHMMs was kept at 0.5 mg/mL and VMF vibration time for 2 h. Therefore, cancer cell killing efficiency can be improved in VMF when co-cultured with UHMMs for a short time. However, cancer cell activity remained above 95% in the control group, which only added UHMMs. There was no significant difference compared with the no-treatment groups. Qualitative detection with Hoechst/propidium iodide fluorescence double staining also yielded results consistent with prior quantitative analysis (Additional file 1: Fig. S8–S10).

A scanning electron microscope (SEM) was used to observe damage to laryngocarcinoma cells after 0.5–4 h of vibration to explore the definite mechanism of cancer cell death caused by UHMMs by generating mechanical force under VMF. The SEM images in Fig. 3i show the destruction of TU212 cells caused by mechanical force with UHMMs (0.5 mg/mL) in VMF (maintaining the maximum value of magnetic intensity at 400 mT and the frequency at 2 Hz). The particles attached to fusiformis cells are UHMMs, and the black holes on the surface of cancer cells show the destruction of the cell membrane by UHMMs. The diameter of the black holes enlarged to ~ 5 μm, and their number increased after 4-h exposure to VMF. Furthermore, lactate dehydrogenase (LDH) leakage corresponds to the extent of cell membrane destruction; 11.03% ± 1.06, 18.37% ± 2.81%, and 27.96% ± 6.04% LDH was released to the cell medium after VMF treatment for 0.5, 2, and 4 h, respectively (Fig. 3h).

Therefore, the mechanical force generated by UHMMs under VMF is an effective in vitro inducer of tumour death. The cancer cell killing efficiency relies on the mechanical force generated by UHMMs in VMF, which destroys cell membrane integrity and releases the contents of the cell.

### Cancer cell killing efficiency of UHMMs mediated by multimode therapy (photothermal, mechanical force, and photodynamic effects)

Next, we used UHMMs@Ce6 (1 mg UHMMs containing 0.115 mg Ce6) to investigate the cancer cell killing efficiency of the combination therapy of the magneto-mechanic force, photothermal, and photodynamic effects under VMF and the 808 nm laser. Furthermore, to prevent the reduction of the photothermal effect caused by UHMM accumulation under a low-frequency vibrating magnetic field, an 808 nm laser was applied before using VMF. We created seven in vitro group experiments: ① RPMI1640 (no treatment), ② Ce6 + laser, ③ UHMMs + VMF, ④ UHMMs + laser, ⑤ UHMMs@Ce6 + laser, ⑥ UHMMs + laser + VMF, and UHMMs@Ce6 + laser + VMF.

First, we maintained the concentration of UHMMs/UHMMs@Ce6 dispersion (dispersed in RPMI1640) at 0.75 mg/mL. Second, TU212 cells were co-cultured with UHMMs and UHMMs@Ce6 solution for 3 h. TU212 cells containing UHMMs or UHMMs@Ce6 solution were irradiated for 10 min with an 808 nm laser (1.08 W/cm^2^) or vibrated with VMF for 2 h. Cell Titer-Glo® assay was used to test the viability of the cancer cells.

**Fig. 4:**
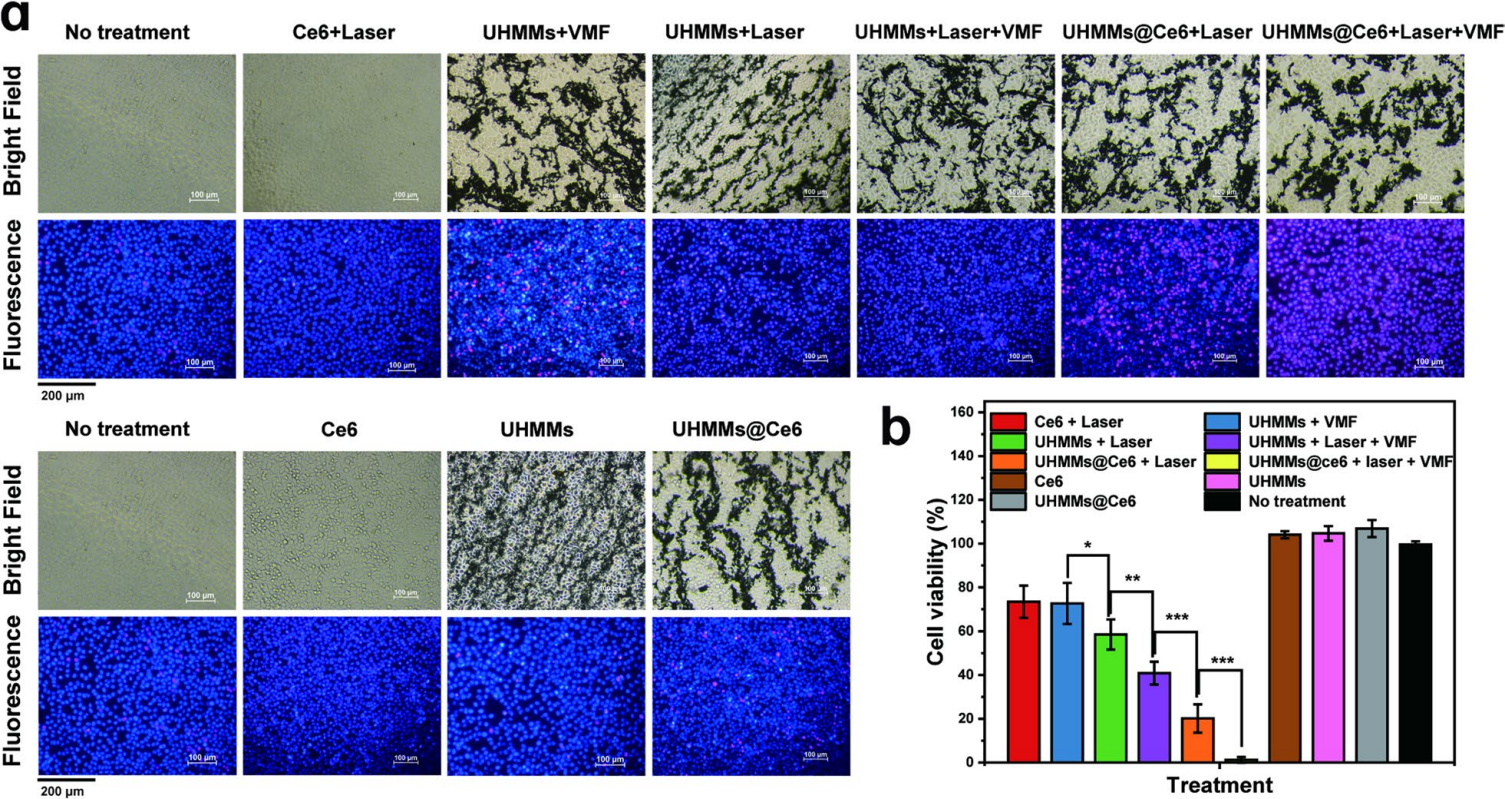
**a** Hoechst/Propidium Iodide fluorescence double-staining images of TU212 cells after treated by single, dual, and multimode in vitro treatment. **b** Cancer cell viability after single, dual, and multimode in vitro treatment

The TU212 cancer cells exhibited more serious destruction with group than the other groups after the VMF and laser combination treatments with UHMMs@Ce6. Approximately 98.83% ± 1.30% of cells were killed after multimode treatment (Fig. 4b), indicating the elimination of almost all cancer cells by magneto-mechanic force, photothermal, and photodynamic effects. However, the UHMMs@Ce6 + laser and UHMMs + laser + VMF groups using two modes to kill TU212 cells also showed moderate injury; 79.87% ± 6.48% of cancer cells were killed under the combination of photothermal and photodynamic effects, and 59.00% ± 5.20% were killed by the photothermal effect and mechanical force. In addition, the variable killing effects (Fig. 4b) for single treatments in groups ②, ③, and ④ were 73.00% ± 7.36%, 72.00% ± 9.40%, and 58.00% ± 6.85%, respectively. The killing efficiency of combined photodynamic and photothermal effects is greater than the sum of a single photodynamic (Ce6) or photothermal (UHMMs) treatment. Thus, photothermal damage could strengthen the photodynamic killing effect. The main reasons that caused efficient destruction are probably attribute to: firstly, the permeability of cancer cells’ membrane was increased by photothermal effect, more ROS generated by Ce6 could enter in weak cancer cells and aggravate the destruction. Secondly, the dark green color of Ce6 may play a little photothermal effect to kill cancer cells. However, the strengthen effect induced by photothermal effect is limited, which will be discussed in more detail in the discussion section. Additionally, compared with the viability of the no-treatment group, cancer cell activity showed no noticeable difference among UHMMs, UHMMs@Ce6, and Ce6 groups without laser or VMF, revealing good UHMMs biocompatibility.

The images of Hoechst/propidium iodide fluorescence double staining qualitative detection indicated the same result among the seven groups (Fig. 4a). The blue and red colours represent live and dead cells, respectively. The number of red cells gradually increases with single, double, and multimode treatments. Hence, the results illustrate that the multimode therapy with UHMMs@Ce6 is a promising therapeutic strategy for tumour damage.

### Photothermal conversion of UHMMs with in vivo 808 nm NIR laser

**Fig. 5:**
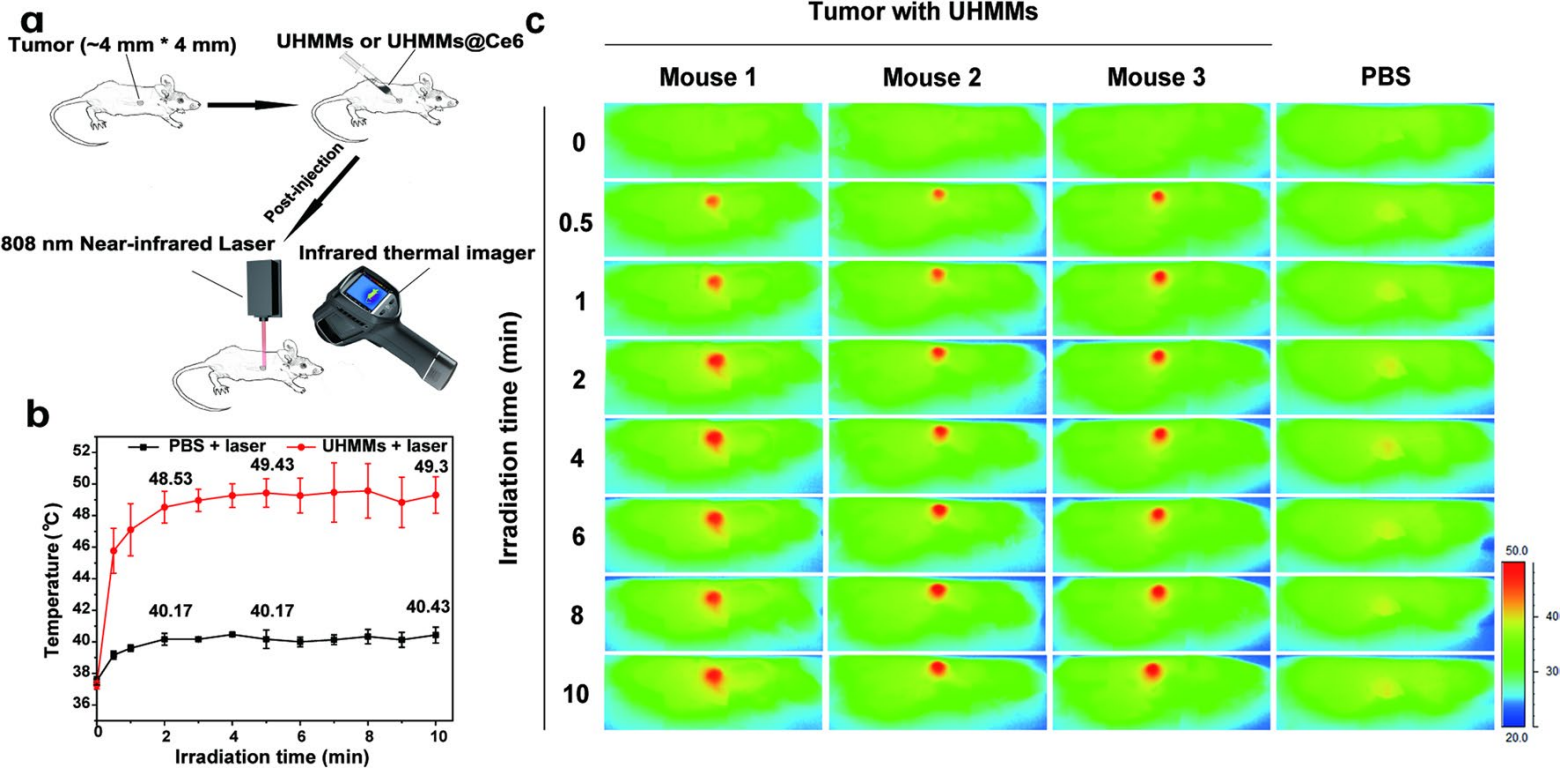
**a** Schematic diagram of the in vivo photothermal convention of UHMMs. **b** Mean tumour temperature profile achieved from the infrared thermal images in Fig. 3a under the 808 nm NIR laser. **c** In vivo infrared thermal images with UHMMs under the 808 nm NIR laser

The good photothermal conversion efficiency of UHMMs under an in vitro 808 nm NIR laser was previously demonstrated. To further identify the photothermal conversion ability of UHMMs for solid tumour treatment applicability, 5 mg/mL of PBS-dispersed UHMMs were directly injected into a tumour by intratumoural injection. Nude mice bearing the tumours were exposed to an 808 nm NIR laser (1.08 W/cm^2^) for 10 min (the tumour site was right under the 808 nm laser apparatus, Fig. 5a) after 12 h of feeding.

Figures 5b, c show the temperature curve and infrared thermal images, respectively. The temperature of the red line, which was injected with UHMMs, showed a significant and rapid increase for the first 2 min under 808 nm laser irradiation, from 37.27 ± 0.25 ℃ to 48.53 ± 1.0 ℃. After 5 min the temperature reached 49.43 ± 0.91 °C and remained steady until 10 min (49.3 ± 1.2 ℃). Notably, the temperature exceeded 42 °C for at least 9.5 min during 10 min of irradiation; thus, the photothermal effect of UHMMs induced by the 808 nm laser effectively damaged the solid tumour. Correspondingly, the temperature of the control group injected by 1 × PBS and treated with the 808 nm laser for 10 min showed a security range (Fig. 5b) and reached 40.43 ℃ ± 0.5 ℃ after 10 min. Thus, the power density and irradiation time of the 808 nm laser in this work are safe.

### In vivo efficiency of laryngocarcinoma solid tumour damage with multimode therapy (photothermal, mechanical force, and photodynamic treatments)

Multimode therapy with UHMMs/UHMMs@Ce6 showed good cancer cell-killing effects using a laser, VMF, or in vitro combination. Therefore, in the current study, eight groups of mice were used for in vivo cancer therapy (Additional file 1: Fig. S11).

Thirty-two tumour-bearing nude mice (tumour volume ≈ 4 × 4 mm) were randomly divided into 8 groups and each group has 4 mice: ① PBS (no treatment), ② Ce6 + laser, ③ UHMMs + VMF, ④ UHMMs + laser, ⑤ UHMMs@Ce6 + VMF, ⑥ UHMMs@Ce6 + laser, ⑦ UHMMs + VMF + laser, and ⑧ UHMMs@Ce6 + VMF + laser. To ensure optimal efficiency in tumour damage, VMF was applied first before treatment with the 808 nm laser.

**Fig. 6:**
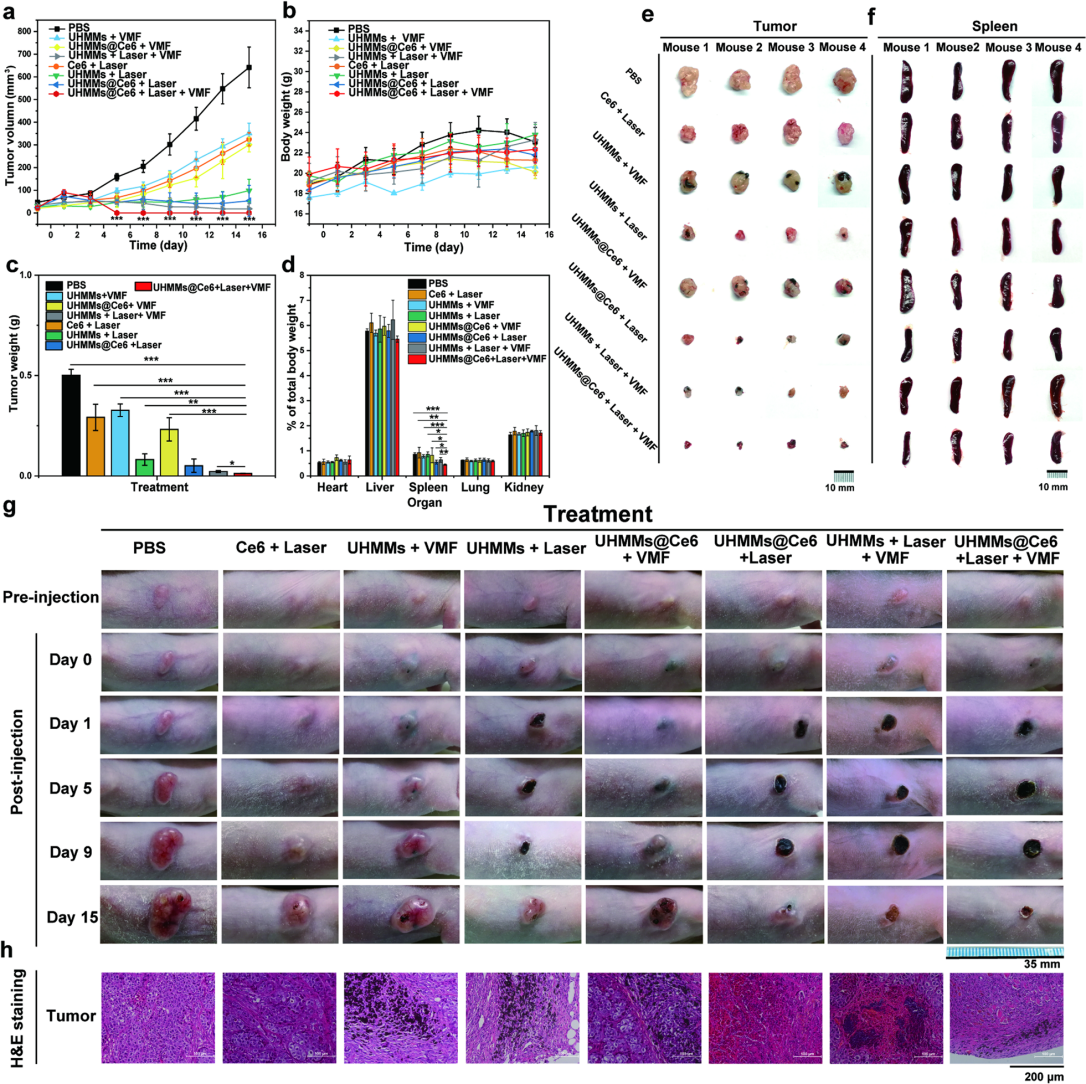
**a** Growth curves of tumours. **b** weight of nude mice over 15 d. **c** weight of tumours after 15 d. **d** organ index of different groups after 15 d. **e** photographs of tumour morphology in the 8 groups after 15 d. **f** photographs of the spleens in the 8 groups after 15 d (photographs of all mice organs are shown in Additional file 1: Fig. S19–S23). **g** representative photographs of mice (photographs of all mice are shown in Additional file 1: Fig. S11–S18). **h** Images of tumour sections by H&E staining in the 8 groups (images of liver and spleen sections by H&E staining are shown in Additional file 1: Fig. S24)

Figures 6a and Additional file 1: S12–S19 show the changes in tumour size with different treatments over 15 days. For group ②, 0.575 mg/mL Ce6 solution (1 × PBS) was injected into the tumour by intratumoural injection; the group was then exposed to 808 nm NIR laser (1.08 W/cm^2^) for 10 min after 12 h of feeding. The tumour growth curve of "Ce6 + Laser" showed was gradually inhibited compared with the PBS group after 7 d. On day 7, the sizes of the tumours in groups ① and ② were 205.22 ± 26.38 and 96.90 ± 24.68 mm^3^, respectively. Fifteen days later, the difference between the tumour sizes of groups ① and ② increased (641.43 ± 89.55 and 324.80 ± 29.45 mm^3^, respectively). On day 15, the weight of the tumour indicated that photodynamics had a negative influence on tumour growth (Fig. 6c). The tumour weights of groups ① and ② were 0.502 ± 0.030 and 0.293 ± 0.066 g, respectively. Notably, the curve of group ② was slightly steeper after 7 d, as Ce6 was easily metabolised in vivo; additionally, photodynamic effects were invalidated by the 808 nm laser during the second treatment.

For groups ③ and ⑤, 5 mg/mL UHMMs or UHMMs@Ce6 dispersion (1 × PBS) was directly injected into the tumour by intratumoural injection; the groups were exposed to VMF (400 mT, 2 Hz) for 2 h after 12 h of feeding. The tumour sizes of these groups showed a growth curve similar to that of the VMF treatment and significantly inhibited tumour growth by magneto-mechanical force five days later. On day 7, the size of the tumours in groups ③ and ⑤ were 117.63 ± 18.51 and 83.14 ± 42.52 mm^3^, respectively. Figure 6a and Additional file 1: Figs. S12, S14, S16 show the significant difference in tumour size between the PBS (group ①, 205.22 ± 26.38 mm^3^) and groups ③ and ⑤. After 15 days of VMF treatment, the tumour volumes of groups ①, ③, and ⑤ were 641.43 ± 89.55, 351.68 ± 44.02 and 300.92 ± 32.02 mm^3^, respectively. On day 15, the weight of the tumour indicated that the magneto-mechanical force induced by VMF significantly inhibited tumour growth (Fig. 6c). The tumour weights of groups ①,③ and ⑤ were 0.502 ± 0.030, 0.327 ± 0.031 and 0.232 ± 0.058 g, respectively. The tumour growth curve initially rose gently but rapidly increased after 7 d for several reasons. First, as the tumour volume increased, the pre-set amount of UHMMs located at a fixed place inside or beside the solid tumour could not transmit the mechanical force to the new-born tumour under VMF; therefore, the inhibition caused by the magneto-mechanical force was weakened, and the tumour growth rapidly increased. These results indicate that the magneto-mechanical force induced by UHMMs and VMF inhibited tumour growth, especially during the early period. However, the magneto-mechanical force should be combined with other therapies to obtain a curative effect in solid tumour therapy because of the weakness of the magneto-mechanical force in the late period.

Group ④ was treated by photothermal irradiation under an 808 nm laser and was very effective against solid tumours. A 5 mg/mL UHMM dispersion (1 × PBS) was directly injected by intratumoural injection, and the group was exposed to an 808 nm laser (1.08 W/cm^2^) for 10 min after 12 h of feeding. Three days after 808 nm laser irradiation, the skin of the tumour side turned dark purple then black (Additional file 1: Fig. S15). The tumour was markedly inhibited and decreased to 48.63 ± 22.49 mm^3^ on day 7 (Fig. 6a). Furthermore, the tumour growth curve was flat and decreased slightly from days 7 to 12. However, the tumour size increased again after 11 days and finally approached 98.50 ± 50.04 mm^3^. The tumour volume of PBS (group ①, 641.43 ± 89.55 mm^3^) was six times larger than that of UHMMs + laser (group ④) (Fig. 6a). On day 15, the tumour weight of group ④ (0.082 ± 0.029 g) was significantly different from that of group ① (Fig. 6c). These results indicate that UHMMs photothermal therapy induced by an 808 nm laser was satisfactorily treated the solid tumour but did not eliminate the tumour, which grew again 11 days later (Additional file 1: Fig. S15).

Tumour growth was further inhibited with dual-mode treatment. Figure 6b and Fig. 6c show that the curative effect was better in group ⑥, which used photothermal and photodynamic treatments under an 808 nm NIR laser than in groups ② or ④ (Additional file 1: Figs. S12, S13, S15, and S17). Similarly, 5 mg/mL UHMMs@Ce6 dispersion (1 × PBS) was directly injected into the tumour by intratumoural injection; the group was exposed to an 808 nm NIR laser (1.08 W/cm^2^) for 10 min after 12 h of feeding. On day 7, the tumour size of group ⑥ (60.60 ± 46.25 mm^3^) was much smaller than that of group ① (205.22 ± 26.38 mm^3^) but close to that of group ④ (48.63 ± 22.49 mm^3^). However, on the last day, the tumour size of group ⑥ (53.46 ± 68.54 mm^3^) was smaller than that of group ④ (98.50 ± 50.04 mm^3^). The tumour weight of group ⑥ (0.051 ± 0.033 g) was also lower than that of group ④ (0.082 ± 0.029 g).

Additionally, the tumour showed better inhibition for combined photothermal and magneto-mechanical forces than for a single treatment. In group ⑦, 5 mg/mL UHMMs dispersion (1 × PBS) was directly injected into the tumour by intratumoural injection for 12 h. Subsequently, the tumour-bearing nude mice were exposed to VMF (400 mT, 2 Hz) for 2 h and irradiated by an 808 nm laser (1.08 W/cm^2^) for 10 min. On day 15, the tumour sizes of groups ③, ④ and ⑦ were 324.80 ± 29.45, 98.50 ± 50.04 and 18.29 ± 22.72 mm^3^ (Fig. 6a, Additional file 1: Figs. S12, S14, S15 and S18). The tumour weight of group ⑦ (0.022 ± 0.005 g) was also significantly lower than that of groups ③ and ④ (0.327 ± 0.031 and 0.082 ± 0.029 g, respectively) (Fig. 6c).

These results indicate that dual-mode treatments of photothermal with photodynamic or magneto-mechanical force have an encouraging curative effect on solid tumour inhibition. Furthermore, the curative effect of tumour inhibition with dual-mode therapy was better than that of a single treatment with UHMMs or UHMMs@Ce6.

Finally, to identify whether the multimode therapy (magneto-mechanic force, photothermal, and photodynamic effects) could better inhibit solid tumours than either dual-mode therapy or single therapy, the tumour-bearing nude mice in group ⑧ were directly injected with 5 mg/mL UHMMs@Ce6 dispersion (1 × PBS) by intratumoural injection. After 12 h of feeding, this group was exposed to VMF (400 mT, 2 Hz) for 2 h vibration and later irradiated for 10 min with an 808 nm NIR laser (1.08 W/cm^2^) immediately. Five days after treatment with VMF and the 808 nm NIR laser, the bulging solid tumours flattened, and the colour turned black (Additional file 1: Fig. S19). These tumours were almost eliminated, and only a small scar appeared on the skin appeared after treatment for 15 d (Fig. 6b and Additional file 1: Fig. S19). Simultaneously, the average weight of the tumours in group ⑧ (0.012 ± 0.001 g) differed significantly from those of the other groups and was extremely significantly different to that of group ① (Fig. 6c). The results indicate that multimode therapy with magneto-mechanic force, photothermal, and photodynamic effects induced by UHMMs with an 808 nm NIR laser and VMF and had an excellent curative effect on solid tumours.

The weights of mice in groups ②–⑧ showed a normal growth curve after treatment with the 808 nm laser and VMF for 15 d (Fig. 6b). However, the weights of mice in groups ③, ⑤, ⑦, and ⑧ treated by VMF were lower than those in group ① (PBS) during the later period. This difference was attributed to the 2-h daily vibration in the VMF apparatus, which negatively influenced the diet and physical health of the mice.

The weight and histological structure of the major organs of the mice showed no significant differences among the different groups, except for the spleen (Fig. 6d, f, Additional file 1: Figs. S20–S24). The spleen is the largest in vivo peripheral immune organ and plays an important role in tumour immunity. Thus, spleen weight was closely correlated with tumour size. The nude mice of group ⑧ treated with the 808 nm laser and VMF had the smallest spleen (0.450% ± 0.027% of total body weight); the spleens of the mice of group ① accounted for 0.864% ± 0.058% of the total body weight. The weights of the tumours in group ① were lower than those in the other groups.

H&E staining of tumours in the groups with different treatments showed significant differences (Fig. 6h). First, the PBS group cells in the solid tumour were plump and had a clear nucleus; further, the structure of cells was completely and tightly packed. However, after a single photodynamic treatment, the cancer cells were loosely packed but maintained a normal morphology. In groups ③ and ⑤, UHMMs/UHMMs@Ce6 were dispersed in the tumour, and the morphology was beside the nanoparticles changed significantly. The gap between the cells was enlarged, and UHMMs/UHMMs@Ce6 destroyed the integrity of the cell structure.

Furthermore, the tumour cells showed ablation and necrosis after treatment with the 808 nm NIR laser because of the high temperature. When dual-mode or multimode treatment was applied, the tumour size decreased, and the morphology of the solid tumour cells also showed similar changes. Black nanoparticles surrounded the cells; the lack of clear boundaries among the cells was due to multiple fragmented cells.

Simultaneously, the morphology and structure of the liver sections in group ⑧ with H&E staining showed no significant differences (Additional file 1: Fig. S25). However, the spleen sections with H&E staining showed little dissimilarity (Additional file 1: Fig. S25). For instance, the spleen weight of the tumour-bearing nude mice in group ① that were injected with PBS due to almost complete tumour elimination was lower than that in group ⑧. The tightness of the spleen cells was higher in groups ⑦ (UHMMs + VMF + Laser) and ⑧ (UHMMs@Ce6 + VMF + laser) and correlated with tumour volume.

Routine blood tests, and hepatic and renal functions tests were carried out at 1st, 7th and 21st days after percutaneous injection with UHMMS@Ce6 dispersion (1 × PBS, 5 mg/mL) in normal ICR mice. These tests included white blood cell (WBC), red blood cell (RBC), haemoglobin (HGB), platelet (PLT), percentage of lymphocytes (LYM %), mean corpuscular haemoglobin (MCH), haematocrit (HCT), plateletcrit (PCT), mean corpuscular volume (MCV)), and total protein (TP), albumin (ALB), globulin (GLOB), alanine aminotransferase (ALT), aspartate aminotransferase (AST), creatinine (Cr), urea (UREA), total bilirubin (TBIL), albumin/globulin (A/G), respectively. The results are showed in Additional file 1: Fig. S26. Most routine blood, hepatic and renal functions tests indexes were normal, and no statistical difference was found compared with the normal group, which further confirmed the safety of UHMMS@Ce6 in vivo.

## Discussions

It is a great goal for many modern medical researchers to search for a simple, efficient, safe, controllable and non-invasive method in cancer treatment. In this work, we used a template-assisted hydrothermal route with a calcination method to prepare a structurally versatile kind of novel urchin-like hollow magnetic microspheres (i.e., UHMMs) in laryngocarcinoma treatment. UHMMs were synthesized by two simple steps from safe and friendly raw materials firstly. Furthermore, compared to other similar works that employ magnetic IONPs [32, 42, 66, 73, 74] commonly with a regular shape (like spheres [18, 61–65]) in cancer treatment, the Fe_3_O_4_ nanorods from UHMMs in our work can not only increase the stimulation of mechanical force to cells [66, 67], but also improve the photothermal conversion ability under near-infrared light because of surface plasmon resonance [68, 69]. The satisfactory structure and better magnetism of UHMMs shows a good application in cancer treatment studies. When loaded with photosensor (i.e., Ce6) in UHMMs, the nanoparticles (UHMMs@Ce6) were well applied in laryngocarcinoma treatment in vitro and in vivo. To the best of our knowledge, this is the first time the drug-loaded microspheres (UHMMs@Ce6) have been employed in cancer treatment by three mode therapies (magneto-mechanic force, photothermal and photodynamic therapy) under a low-frequency vibrating magnetic field and an 808 nm near-infrared laser.

In the past research work, photothermal therapy with NIR laser and magnetic IONPs was an effective strategy in tumor treatment. However, two obstacles hindered its performance: insufficient penetration of the NIR laser [34–38], and poor photothermal conversion ability with IONPs [44]. Thus, many researchers used combination therapies like PTT-PDT [28], PTT-SDT (sonodynamic therapy) [57, 58] or PTT/PDT-chemistry [59, 60] to improve the efficacy of cancer treatment. However, the mentioned obstacles were easily tackled in the current study using vibrating magnetic field and urchin-like Fe_3_O_4_ nanoparticles. The advantages of magneto-mechanic force and low frequency vibrating magnetic field included: (1) Low frequency dynamic or alternating magnetic field is very easy to build and has little effect on the human body. (2) Magnetic IONPs could display movement, oscillate or rotate under low-frequency vibrating magnetic fields and generate mechanical force to stimulate cancer cells to induce cell apoptosis or death [44–47]. (3) Compared with NIR, the magnetic field could penetrate deeper into tissues and affect magnetic particles’ behaviour inside tissues, which could stimulate deeper into the tumour for destroying it. (4) The vibrating magnetic field used in this magneto-mechanical actuation technique is usually only a few tens of Oersteds with a frequency of less than 100 Hz [44], compared to traditional high frequencies alternating magnetic field. The low frequency and low magnetism intensity of VMF make it safer with ease in handling. (5) The mechanical force for manipulating tumour evolution only requires a pico-Newton (*pN*) level [55, 56].

Additionally, very few groups except for ours have the expertise to conduct this type of research using low frequency vibrating magnetic fields. Therefore, UHMMs@Ce6 synthesized in the present work could combine the advantages of both therapies and deliver a photosensor to a target position to kill cancer with ROS production, simultaneously. The results of laryngocarcinoma treatment in vitro and in vivo are encouraging. Moreover, the solid tumors were almost eliminated by 3 modes combined therapy in tumor-bearing nude mice.

There were some interesting phenomena during the experiments in vitro/vivo that deserve discussion. The curative effect of cancer cell killing and tumor inhibition applied dual/multimode therapy was better than the summary of single treatment with different treatment. For instance, this work showed a synergistic effect when using PTT&PDT in cancer treatment, which was attributed to (1) the higher toxicity of UHMMs@Ce6 than UHMMs towards cancer cells because the loaded Ce6 had a negative charge. (2) The photothermal conversion efficiency on the peak of urchin-like Fe_3_O_4_ particles could enhance Ce6 to produce ROS under an 808 nm NIR laser. (3) The permeability of cancer cells’ membrane could be increased by photothermal effect, more ROS generated by Ce6 could enter into weak cancer cells and aggravate the destruction. (4) The dark green color of Ce6 might play a little photothermal effect to kill cancer cells. However, the synergistic effect induced by 808 nm laser is limited, because the wavelength of 808 nm laser is not an ideal one to trigger Ce6 to produce high amount of ROS. Therefore, to elevate the synergistic effect with PTT&PDT with different wavelength near-infrared laser will surely be a worthy and effective way to kill the cancer cells.

In a continuation of this work of future research studies are planned and will include improvements in the following two aspects: (1) Reducing the diameter of UHMMs and modifying hydrophilic groups on the surface of UHMMs to improve their dispersion inside the solid tumor. (2) Selecting new drugs like chemotherapeutic drugs to load in UHMMs that can reside longer in vivo. Additionally, the apparatus of VMF could be made smaller and more portable. The hand-held size of it can be realized to facilitate the treatment of superficial cancer in future.

## Conclusions

In summary, UHMMs with strong magnetism and low cytotoxicity were synthesised for cancer therapy. Upon exposure to a low-frequency dynamic magnetic field, UHMMs efficiently damage laryngocarcinoma cells and inhibit mouse tumour growth through a magneto-mechanical force. UHMMs are also excellent photothermal reagents and drug carriers because of their black colour and hollow structure. After Ce6 molecules were incorporated into UHMMs, the UHMMs@Ce6 obtained under 808 nm laser irradiation exhibited strong photothermal and thermal effects that improved the photodynamic killing effect on cancer cells and more efficiently inhibited tumour growth. When UHMMs@Ce6 were simultaneously exposed to the dynamic magnetic field and the laser, the cancer cells were almost completely killed. In addition, mouse tumours could not be detected, because the cells and tumour tissue were affected by the synergy of the mechanical force, photothermal, and photodynamic effects. This work is the first report of the combination of magnetic microspheres with unique structures and cancer treatment, and this may have great potential as a platform to treat various tumours, especially superficial solid tumours.

## Methods

### Chemicals

Monodisperse micrometre-sized carboxyl-functionalised polystyrene particles were purchased from Polysciences (520 nm diameter), ferrous sulfate heptahydrate was obtained from Sinopharm, and Chlorin e6 was purchased from the Shanghai Yuanye Biotechnology Limited Company.

Instrumentation: TEM images were obtained using a JEM 1230 transmission electron microscope (120 kV). SEM imaging and element mapping were conducted using a Hitachi S-3400 N and Gemini SEM 500 field emission scanning electron microscope (30 kV). UV − vis spectra were measured using a Shimadzu spectrophotometer. Zeta potentials were measured using a Malvern Zetasizer. Powder XRD was performed using a Rigaku Ultima IV X-ray diffractometer diffractometer. The magnetisation saturation rate was measured using a Lake Shore 7404 vibrating sample magnetometer. The activity and fluorescence of the cells were recorded using a SpectraMax M5 Multifunctional microplate reader.

### Synthesis of UHMMs particles

UHMMs were fabricated through a two-step process: First, 100 μL of carbonylated polystyrene microsphere original solution was added to 20 mL of ferrous sulfate solution (0.116 g FeSO_4_7H_2_O dissolved in 35 mL deionised water). Next, the mixed liquid was transferred to a Teflon-lined autoclave (25 mL) and heated to 120 °C for 10 h to synthesise the precursor of UHMMs containing Fe_2_O_3_ nanorod shells and PS cores. The precursor particles were then collected after natural cooling to room temperature and washed twice with deionised water and absolute ethanol. Later, the particles were harvested after drying at 75 °C for 8 h in a drying box.

Because the polystyrene microspheres could be removed under hyperthermia with an Ar/H_2_ (5%) environment and used to transform Fe_2_O_3_ to Fe_3_O_4_, Fe_2_O_3_@PS was placed in a tube furnace under Ar/H_2_ (5%) and heated to 320 °C at 5 °C /min ramps from room temperature for 80 min. The synthesised product was finally obtained after natural cooling to room temperature.

### Synthesis of UHMMs@Ce6 particles

The synthesised UHMMs (2 mg) were dispersed in 1 mL absolute ethanol and mixed with 1 mL Ce6 solution (Ce6 powder was dissolved in dimethyl sulfoxide to form a 20 mg/mL solution and prepared at a concentration of 1 mg/mL with absolute ethanol. Next, the mixed solution was placed in a dark table for 24 h at 220 rpm. The UHMMs@Ce6 particles were isolated by magnetism and washed with 1 × PBS after dispersion.

### VMF apparatus

The VMF apparatus developed by our group was used in this study [44]. Briefly, two NdFeB permanent magnets (T = 6800 G) were fixed on an aluminium sheet; the distance between the two magnets was 4.5 cm. The two permanent magnets moved up and down simultaneously with a fixed distance of 3 cm and motor disk rotation. In the treatment position, the change in the magnetic field was transformed into variations in the UHMM mechanical force. When the turntable reaches its lowest position, the top and bottom of the magnet show the maximum and minimum magnetism values for the treatment position, respectively. Similarly, when the turntable reaches the highest position, the bottom and top of the magnet show the maximum and minimum magnetism values for the treatment position, respectively. The vibration frequency of the magnets was adjusted by the motor rotation speed. In this work, the vibration frequency of the VMF apparatus was 2 Hz, and the magnetic field intensity was 400 mT.

### Cancer cell killing with mechanical force induced by UHMMs during VMF

TU212 cells were seeded in three wells (positions: B2, G2, and G11) (each well contained 1 × 10^4^ cells) in a 96-well plate. In this plate, well B2 used VMF treatment, whereas the others included no treatment. The UHMMs were dispersed in RPMI 1640 culture medium (without fetal bovine serum and phenol red) to form 0.3, 0.5 and 1.0 mg/mL dispersion after a 24-h culturing interval. The medium in wells B2 and G11 was replaced with UHMM dispersions at different concentrations. The medium in well G2 was replaced with an RPMI 1640 culture medium alone as a no-treatment control (150 µL per well). Subsequently, the 96-well plate was co-cultured in a 5% CO_2_ incubator at 37 °C for 1, 3, and 6 h and placed between the two magnets of the VMF apparatus. Well B2 was exposed to VMF for 0.5, 2, and 4 h. The vibration frequency of the VMF was maintained at 2 Hz and 400 mT. The 96-well plate containing the cancer cells was the incubated in a 5% CO_2_ incubator at 37 °C for 2 h before conducting the cell viability assay. Each experiment was repeated five times.

### Cancer cell killing with photothermal/photodynamic effects induced by UHMMs/UHMMs@Ce6 using the 808 nm NIR laser

Similarly, TU212 cells were seeded in three wells (B2, G2, and G11) (each well contained 1 × 10^4^ cells) of a 96-well plate. The B2 well used laser treatment, whereas the others were left untreated. The UHMMs/UHMMs@Ce6 were dispersed in RPMI-1640 culture medium to form 0.5, 0.75, and 1.0 mg/mL dispersion after culturing for 24 h. The medium in wells B2 and G11 was replaced by UHMMs/UHMMs@Ce6 dispersion at different concentrations, and the medium in well G2 was replaced with RPMI-1640 culture medium alone as a no-treatment control (150 µL per well). Subsequently, the 96-well plate was co-cultured in a 5% CO_2_ incubator at 37 °C for 3 h, followed by placement under the 808 nm NIR laser apparatus. The B2 well was exposed to the NIR laser for 5, 10, and 15 min. The power density of the 808 nm NIR laser was ~ 1.08 W/cm^2^ (power ≈ 0.38 W), the spot area was 1 cm × 0.35 cm, and the distance between the plate and laser source was 10 cm. The experiment was conducted in a dark environment after adding the UHMMs@Ce6 dispersion. Cancer cells in the 96-well plate were incubated in a 5% CO_2_ incubator at 37 °C for 2 h before conducting the cell viability assay. Each experiment was repeated five times.

### Cancer cell damage induced by UHMMs exposed to both VMF and NIR lasers

Similarly, TU212 cells were seeded in three wells (B2, G2, and G11) (each well contained 1 × 10^4^ cells) of a 96-well plate. The B2 well used laser and VMF treatment, whereas the others had no treatment. The UHMMs or UHMMs@Ce6 were dispersed in RPMI-1640 culture medium to form a 0.75 mg/mL dispersion after culturing for 24 h. The mediums in well B2 and G11 were replaced by 0.75 mg/mL UHMMs/UHMMs@Ce6 dispersion, and the medium in well G2 was replaced by RPMI-1640 culture medium as a no-treatment control (150 µL per well). Subsequently, the 96-well plate was co-cultured in a 5% CO_2_ incubator at 37 °C for 3 h and placed under the VMF or 808 nm NIR laser apparatus as described above. The B2 well was exposed to the NIR laser for 10 min and treated with VMF for 2 h. The power density of the 808 nm NIR laser and vibration frequency of the VMF treatment were the same as mentioned above. The experiment was conducted in a dark environment after adding UHMMs@Ce6 dispersion. The cancer cells in the 96-well plate were incubated in a 5% CO_2_ incubator at 37 °C for 2 h before conducting the cell viability assay. Each experiment was repeated five times.

### CellTiter-Glo® assays were used to detect the activity of the cancer cells

The CellTiter-Glo® luminescent assay kit is a homogeneous method for detecting the number of living cells by quantitative measurement of ATP, which is an indicator of the metabolism of living cells. The addition of reagents causes the cells to lyse and to produce a luminous signal proportional to the amount of ATP. Therefore, living cells are measured indirectly by the amount of ATP.

The buffer solution in the CellTiter-Glo® kit was mixed with the powder to obtain a homogeneous solution and stored at 4 °C. One hundred microliters of the mixed solution was replaced with the culture medium of B2, G2, and G11 in 96-well plates after VMM or laser treatment. The reaction time was maintained for 10 min at room temperature, and a SPECTROMAX M5 multifunctional microplate reader was used to detect bioluminescence. The activity of cells in each group was calculated using Eq. (4):

4 $$Activity\,of\,cells \%=\frac{luminous\,value\,of\,treatment\,group}{luminous\,value\,of\,no\,treatment\,group}\times 100$$

### Apoptosis and necrosis of cancer cells were detected by Hoechst 33342 and propidium iodide (PI) double staining assays

Hoechst 33342 is a blue fluorescent dye that penetrates the cell membrane, releases strong blue fluorescence after being embedded in double-stranded DNA, and has no significant cytotoxicity. The blue fluorescence of apoptotic cells was stronger than that of normal cells. PI is also a nuclear staining reagent that can stain DNA or RNA and produces red fluorescence when combined with nucleic acids. However, PI does not penetrate the cell membrane and or stain normal or apoptotic cells with intact cell membranes. For necrotic cells or late apoptotic cells, the loss of the integrity of the cell membrane allows red staining with PI. According to the above-mentioned principles, apoptotic, necrotic, and normal cells can be distinguished under a fluorescence microscope.

First, 5 μL Hoechst 33342 dye solution and 15-μL PI dye solution were added to a 1-ml buffer solution and evenly mixed. The mixed dye solution (200 μL) replaced the culture medium of B2, G2, and G11 in 96-well plates after VMT or laser treatment and was incubated at 4 °C for 30 min. After staining, the cells were washed once with PBS, and fluorescence was observed under a fluorescence microscope.

### LDH release assay

The integrity of the cell membrane was destroyed by the mechanical force induced by UHMMs under the VMF treatment; abundant cell contents and intracellular enzymes were released into the extracellular space, including lactate dehydrogenase (LDH). The absorption intensities at 490 nm of cell culture media containing the LDH test working solution were measured, and the LDH leakage was calculated using formula (5):

5 $$LDH\,leakage \left(\%\right)=\frac{absorbance\,of\,treatment\,cells-absorbance\,of\,control\,cells}{absorbance\,of\,maximal\,enzyme\,activity\,cells-absorbance\,of\,control\,cells}\times 100$$

Similarly, TU212 cells were seeded in three wells (positions: B2 (cells + UHMMs + Laser for VMF treatment group), B11 (cells for maximum enzyme activity control well), E7 (no cells but culture medium for blank background group), G2 (cells only for untreated group), and G11 (cells + UHMMs for the sample control group)); each well contained 1 × 10^4^ cells in a 96-well plate. The UHMMs were dispersed in RPMI-1640 culture medium (without fetal bovine serum and phenol red) to form a 0.5 mg/mL dispersion. After culturing for 24 h, the medium in wells B2 and G11 was replaced by UHMM dispersion with 0.5 mg/mL UHMMs; the medium in wells B11, G2, and E7 was replaced by RPMI-1640 culture medium (150 µL per well). Subsequently, the 96-well plate was co-cultured in a 5% CO_2_ incubator at 37 °C for 1, 3, and 6 h and placed between the two magnets of the VMF apparatus. The B2 well was exposed to VMF for 0.5, 2, and 4 h. The VMF vibration frequency was maintained at 2 Hz, and the strength was set at 400 mT. The cancer cells in the 96-well plates were incubated in a 5% CO_2_ incubator at 37 °C for 2 h, and 100 µL LDH release reagent of LDH release assay kit was added to B11. The 96-well plate was continually incubated in a 5% CO_2_ incubator at 37 °C for 1 h.

The 96-well plate was centrifuged at 1400 rpm for 5 min. The 120 μL of supernatant from each well was transferred to a new 96-well plate, and 60 μL of LDH detection reagent was added to the supernatant and thoroughly mixed. The reaction time was set to 30 min in a dark environment at room temperature, and the optical density (OD) at 490 nm was measured using a SPECTROMAX M5 multifunctional microplate reader.

### Morphology analysis of cells before and after VMF treatment with UHMMs using SEM

The cell morphology analysis method was improved from a previous report. TU212 cells were seeded in three 6-well plates (each well contained 1 × 10^5^ cells placed around the cell climbing film (d = 2 cm)). After culturing for 24 h, the medium in the first and second plates was replaced with 2 mL UHMM dispersion in each well (0.5 mg/mL dispersed in RPMI-1640 culture medium (without fetal bovine serum and phenol red)); the medium of the third plate was replaced with 2 mL pure RPMI-1640 culture medium (without fetal bovine serum and phenol red). Next, the first 6-well plate cells were exposed to VMF for 0.5, 2, or 4 h (the vibration frequency and magnetism density of VMF were kept consistent with the experiments before) after 3 h of incubation. Before SEM examination, the cell climbing films were fixed with 2.5% glutaraldehyde at 4 °C for 12 h, washed (twice with 1 × PBS and once with deionised water); the residual water was absorbed with absorbent paper. The cell climbing films were then cryopreserved at − 80 °C for 2 h and placed in a vacuum freeze-drying apparatus (Christ Alpha 1–2 LD plus, Martin Christ Gefriertrocknungsanlagen) for vacuum freeze-drying. The drying cell climbing films were coated with gold using a high vacuum ion sputtering instrument (Hitachi E-1010) to improve conductivity before SEM observation.

### In vivo cancer therapy

Animal experiments were performed following the guidelines of the University of Tongji Institutional Animal Care and Use Committee.

TU212 cells dispersed in PBS (100 µL, 5 × 10^6^ cells mL^−1^) were subcutaneously injected into the flank of nude mice (n = 32), which were fed for 5 days to harvest tumour-bearing nude mice; the tumour sizes were maintained at approximately 4 mm × 4 mm. The mice were randomly divided into eight groups of four mice: ① PBS (no treatment), ② Ce6 + laser, ③ UHMMs + VMF, (4) UHMMs@Ce6 + VMF, ⑤ UHMMs + laser, ⑥ UHMMs@Ce6 + laser, ⑦ UHMMs + laser + VMF, and ⑧UHMMs@Ce6 + laser + VMF. Additional file 1: Figure S12 presents the experimental details. Because the UHMM size surpasses 500 nm and there are no target molecules modified on the surface, UHMMs might block blood vessels and hardly reach the tumour site through intravenous injection. Therefore, UHMMs or UHMMs@Ce6 were injected into solid tumours directly through intratumoural injection; and the concentration of UHMMs or UHMMs@Ce6 dispersion was maintained at 5 mg/mL in 1 × PBS. The Ce6 solution was maintained at 575 μg/mL in PBS.

In all animal experiments, the strength and frequency of the VMF were maintained at 400 mT and 2 Hz, respectively, and the power density of the 808 nm NIR laser was maintained at 1.08 W/cm^2^, respectively.

For group ① (a no-treatment control group), 100 μL of (1 × PBS) solution was injected into the tumour by intravenous injection. Mice weight and tumour volume were recorded every 2 d for 15 d.

For group ② (a single photodynamic treatment group), 100 μL of 575 μg/mL Ce6 solution (1 × PBS) was injected into the tumour at the same site as group ① after 12 h of feeding; the nude mice were then exposed to the 808 nm NIR laser apparatus for photodynamic treatment. The distance between the tumour and the laser source was set at 10 cm, and the irradiation time was maintained at 10 min. Each mouse was treated with an 808 nm NIR laser once a week for 15 d.

For groups ③ and ④, 100 μL of 5 mg/mL UHMMs, or UHMMs@Ce6 dispersion (1 × PBS) was injected into the tumour at the same site as in group ① to identify the mechanical force damage induced by VMF. After 12 h, the nude mice were exposed to the low-frequency VMF apparatus for magnetic mechanical force treatment; each mouse was treated with VMF every day for 15 d.

For group ⑤, 100 μL of 5 mg/mL UHMM dispersion (1 × PBS) was injected into the tumour at the same site as in group ① to identify the photothermal damage induced by the 808 nm laser. After 12 h of feeding, the nude mice were exposed to the 808 nm laser apparatus for photothermal treatment, and the irradiation conditions were maintained as with group ②. Each mouse was treated with an 808 nm laser once a week for 15 d.

For group ⑥, 100 μL 5 mg/mL UHMMs@Ce6 dispersion (1 × PBS) was injected into the tumour at the same site as in group ① to identify the damage from the dual-mode therapy with photothermal and photodynamic treatments induced by the 808 nm laser. After 12 h of feeding, the nude mice were exposed to the 808 nm laser apparatus for photothermal and photodynamic treatment; the irradiation conditions were maintained as with group ②. Each mouse was treated with an 808 nm laser once a week for 15 d.

For group ⑦, 100 μL 5 mg/mL UHMM dispersion (1 × PBS) was injected into the tumour at the same site as in group ① to identify the damage from the dual-mode therapy with magnetic mechanic force and photothermal treatment induced by the VMF 808 nm laser. After 12 h of feeding, the nude mice were exposed to the low-frequency VMF apparatus for magnetic mechanical force treatment at and treated with the 808 nm laser as described above. Each mouse was treated with the 808 nm laser once a week but with VMF every day for 15 d.

For group ⑧, 100 μL of 5 mg/mL UHMMs@Ce6 dispersion (1 × PBS) was injected into the tumour at the same site as in group ①; the tumour-bearing nude mice were treated by multimode therapy by mechanical force, photothermal, and photodynamic treatments induced by VMF and the 808 nm laser. After 12 h of feeding, the nude mice were exposed to the low-frequency VMF apparatus for magnetic mechanical force treatment and treated with the 808 nm laser as described above. Each mouse was treated with the 808 nm laser once a week but with VMF every day for 15 d.

The morphologies and volume of the solid tumour and weight of the mice were recorded at 2 d intervals during treatment using a digital camera, calliper, and balance. Tumour volumes (V) were calculated based on $V = 1/2 \times L \times W^{2}$, where *L* is the length of the solid tumour, and *W* is the width.

Tumours bearing nude mice were sacrificed, and the tumours and main organs (including the heart, liver, spleen, lung, and kidney) were resected (for the tumours that disappeared, the skin at the original tumour site was resected), weighed, and fixed in 4% paraformaldehyde solution at 4 °C for 48 h. The tumours, livers, and spleens of all the nude mice were stained with H&E to observe the histopathological differences on day 15.

### In vivo photothermal conversion of UHMMs

To reduce the sacrifice of nude mice, three tumour-bearing nude mice originated from groups ④ and three from group ①. Similarly, 100 μL of 5 mg/mL UHMMs dispersion (1 × PBS) was directly injected into the tumour by intratumoural injection. Tumour-bearing nude mice were exposed to an 808 nm laser (1.08 W/cm^2^) for 10 min (the tumour site was right under the laser apparatus (Fig. 5a) after 12 h of feeding. The infrared thermal imager was applied to take photos and to simultaneously record the temperature of the tumour site at 30-s intervals. The mice in the control group were injected with 100 μL 1 × PBS; the other operations followed those of group ④.

### In vivo routine blood and biochemistry tests

16 normal healthy ICR mice were divided into 4 groups randomly. The mice of first 3 groups were injected with 100 uL of UHMMs@Ce6 dispersion (5 mg/mL, 1 × PBS) percutaneously at 1st, 7th and 21st day respectively. The mice of last group had no treatment. The routine blood and biochemistry tests were measured by extracting the blood at 21st day.

### Statistical analysis

The results are expressed as the mean ± standard deviation of each sample. All cellular and animal experiments were independently repeated for at least three times. The results were analysed with Student's t-test or one-way analysis of variance (ANOVA) using SPSS software (Version 24, IBM, USA). *, ** and *** indicate the level of significance P < 0.05, 0.01 and 0.001, respectively.

## Supplementary Information

**Additional file 1.** Additional figures.

## Data Availability

The data that support the findings of this study are available from the corresponding author upon request.
