# Supplementary material for: Urchin-like magnetic microspheres for cancer therapy through synergistic effect of mechanical force, photothermal and photodynamic effects
Source: J Nanobiotechnology. 2022 May 12;20:224. doi: 10.1186/s12951-022-01411-y (PMC9097396; doi:10.1186/s12951-022-01411-y)
Supplement: Supplementary file 1 — Additional file 1. Additional figures. [file 12951_2022_1411_MOESM1_ESM.docx]

**Additional file 1**

**Title**

**Urchin-Like Magnetic Microspheres for Cancer Therapy through Synergistic Effect of Mechanical Force, Photothermal and Photodynamic Effects**

**Authors**

Kai Wu^1,2^**^†^**, Ali Mohsin^1^**^†^**, Waqas Qamar Zaman^3^, Zefei Zhang^2^, Wenyan Guan^4^, Maoquan Chu^2^*, Yingping Zhuang^1^ and Meijin Guo^1^*

**Results**

**Figure S1.** (a, b, c) Morphology of Fe_2_O_3_@PS with different heating time during hydrothermal reaction (120℃, 0.012 mol/L Fe^2+^); (d, e, f) Morphology of UHMMs with different heating temperature during hydrothermal reaction (10h, 0.012 mol/L Fe^2+^); (g, h, i) Morphology of UHMMs with different with different concentration of Fe^2+^ during hydrothermal reaction (120℃, 10h). All scale bar are of 200 nm.

**Figure S2.** (a,h)Morphology of Fe_2_O_3_@PS before template removing in tube furnace; (b-e) Morphology of UHMMs with different heating temperature for 90 min in tube furnace; (i-l) Morphology of UHMMs with different heating time at 360 ℃ in tube furnace.

**Figure S3.** (a) In vitro toxicity of UHMMs with L929 cells. (b) In vitro toxicity of UHMMs@Ce6 with L929 cells.

**Figure S4.** (a) Distribution of UHMMs in deionized water with DLS measurement. (b) Distribution of UHMMs in 1 × PBS with DLS measurement.

**Figure S5.** Low-frequency vibrating magnetic field apparatus.

**Figure S6.** Hoechst/Propidium Iodide fluorescence double staining images of TU212 cells after treated by 808 nm NIR laser for 10min with 0.5 mg/mL, 0.75 mg/mL and 1.0 mg/mL UHMMs dispersion.

**Figure S7.** Hoechst/Propidium Iodide fluorescence double staining images of TU212 cells after treated by 808 nm NIR laser for 5min, 10min and 15min with 0.75 mg/mL UHMMs dispersion.

**Figure S8.** Hoechst/Propidium Iodide fluorescence double staining images of TU212 cells after treated by VMF for 2h with 0.3 mg/mL, 0. 5 mg/mL and 1.0 mg/mL UHMMs dispersion.

**Figure S9.** Hoechst/Propidium Iodide fluorescence double staining images of TU212 cells after treated by VMF for 0.5 h, 2 h and 4 h with 0.5 mg/mL UHMMs dispersion.

**Figure S10.** Hoechst/Propidium Iodide fluorescence double staining images of TU212 cells after treated by VMF for 2 h with 1h, 3 h and 6 h incubation time.

**Figure S11.** Schematic diagram of animal experiments with different treatments by 808 nm laser/VMF.

**Figure S12.** Photographs of tumors morphology in PBS group (group ①) which had no treatment during 15 days.

**Figure S13.** Photographs of tumors morphology in "Ce6 +laser" group (group ②) which was only treated by photodynamic during 15 days.

**Figure S14.** Photographs of tumors morphology in "UHMMs + VMF" group (group ③) which was only treated by magneto-mechanic force during 15 days.

**Figure S15.** Photographs of tumors morphology in "UHMMs + Laser" group (group ④) which was only treated by photothermal therapy during 15 days.

**Figure S16.** Photographs of tumors morphology in "UHMMs@Ce6 + VMF" group (group ⑤) which was only treated by magneto-mechanic force during 15 days.

**Figure S17.** Photographs of tumors morphology in "UHMMs@Ce6 + Laser" group (group ⑥) which was treated by photothermal and photodynamic during 15 days.

**Figure S18.** Photographs of tumors morphology in "UHMMs + VMF + Laser" group (group ⑦) which was treated by magneto-mechanic force and photothermal during 15 days.

**Figure S19.** Photographs of tumors morphology in "UHMMs@Ce6 + VMF + Laser" group (group ⑧) which was treated by magneto-mechanic force, photothermal and photodynamic during 15 days.

**Figure S20**. Photographs of heart at 15^th^ day.

**Figure S21**. Photographs of liver at 15^th^ day.

**Figure S22.** Photographs of spleen at 15^th^ day.

**Figure S23.** Photographs of lung at 15^th^ day.

**Figure S24.** Photographs of kidney at 15^th^ day.

**Figure S25**. Images of spleen and liver sections after H&E staining at 15^th^ day.

**Figure S26**. (a) Routine blood analysis of ICR mice at 1^st^, 7^th^ and 21^st^ day after injected with UHMMs@Ce6 dispersion (1 × PBS, 5mg/mL); (b) Hepatic and renal functions analysis of ICR mice at 1^st^, 7^th^ and 21^st^ day after injected with UHMMs@Ce6 dispersion (1 × PBS, 5mg/mL).
